# Auditory Perception and Psychosocial Well-Being in Long-Term Cochlear Implant Users

**DOI:** 10.3390/audiolres16030083

**Published:** 2026-05-28

**Authors:** Kadriye Guney, Ozlem Topcu, Patrizia Mancini, Hilal Dincer D’Alessandro

**Affiliations:** 1 Department of Audiology, Istanbul University-Cerrahpaşa, 34320 Istanbul, Turkey; kadriyeguney98@gmail.com; 2 Department of Audiology, Hacettepe University, 06100 Ankara, Turkey; ozlem.topcu@hacettepe.edu.tr; 3 Department of Sense Organs, Sapienza University of Rome, 00185 Rome, Italy; p.mancini@uniroma1.it

**Keywords:** cochlear implant, long-term outcomes, psychosocial well-being, learned helplessness, speech perception in noise, auditory perception

## Abstract

**Background/Objectives**: This study investigated auditory perception and psychosocial well-being in long-term cochlear implant (CI) users, with a particular focus on the effects of auditory (re)habilitation on learned helplessness and speech-in-noise perception, representing everyday listening performance. **Methods**: Thirty CI users and thirty peers with typical hearing (TH) participated in the study. Speech perception was assessed using the Hearing in Noise Test (HINT) and the Matrix Test in both quiet and noisy listening conditions. Psychosocial status was evaluated using the Learned Helplessness Scale (LHS), the Beck Depression Inventory (BDI), and the Beck Anxiety Inventory (BAI). Perceived hearing quality was evaluated using the Hearing Implant Sound Quality Index (HISQUI). **Results**: CI users showed significantly poorer speech perception performance than TH participants (*p* < 0.05), whereas between-group psychosocial outcomes, including LHS, BDI, and BAI scores, did not differ significantly (*p* > 0.05). Positive correlations were observed between Matrix and HINT scores in quiet and noisy conditions. Positive associations were also observed between CI hearing thresholds and HINT/Matrix results in noisy conditions. Within the prelingually deaf CI subgroup, age at implantation was correlated with CI thresholds, as well as with speech perception performance across both tests (*p* < 0.05). **Conclusions**: Although CI users showed significantly poorer speech perception performance, their levels of learned helplessness, depression, and anxiety were comparable to those of their TH peers. These results suggest auditory benefits following long-term CI rehabilitation, while psychosocial status appears to be within a typical range despite persistent listening difficulties in daily life.

## 1. Introduction

Hearing loss is a significant risk factor for mental health issues, with severe-to-profound hearing loss having the greatest potential negative impact. Indeed, such significant levels of hearing loss may negatively affect psychosocial and cognitive development. Specifically, prelingual hearing loss may negatively affect social, emotional, and cognitive development in children when it is accompanied by delayed or insufficient access to language, whether spoken or signed [1]. Likewise, postlingual hearing loss may lead to anxiety–withdrawal, reduced social participation, and a higher risk of depressive symptoms [2]. Limitations in communication and difficulties in expressing needs may further contribute to social isolation and mental health problems. Indeed, children with hearing impairment have been shown to be more likely to exhibit symptoms of depression, anxiety, and learned helplessness (LH) than children with typical hearing (TH) [3].

Cochlear implantation has been proven to be an effective treatment for bilateral severe-to-profound hearing loss, particularly when performed early in life or after a minimal duration of auditory deprivation [4]. For example, children who receive a cochlear implant (CI) at an early age have been shown to acquire language skills comparable to those of their TH peers [5]. However, even when language skills are comparable, some CI users continue to experience delays in social competence and may exhibit anxiety–withdrawal and anger–aggression behaviors [6]. Traditionally, CI efficacy has been evaluated using audiological measures such as hearing thresholds and speech perception performance [7]. However, successful (re)habilitation extends beyond auditory–linguistic performance and encompasses mental health, subjective well-being, and social competence [8].

In recent years, researchers have increasingly focused on the psychosocial effects and implications of cochlear implantation, including both its benefits and challenges [9]. Nearly half of individuals with severe-to-profound hearing loss, particularly those with postlingual deafness, have been found to experience mental health difficulties prior to cochlear implantation [10]. Cochlear implantation, in turn, (re)establishes functional hearing and significantly improves quality of life in deaf or hard-of-hearing individuals [11]. However, CI users are known to struggle in complex listening tasks involving background noise, which is usually unavoidable in daily auditory/verbal communication settings [12,13,14]. Such everyday auditory difficulties may have psychological consequences that extend beyond communication challenges [2,3,10]. Repeated experiences of listening difficulties [5] may trigger frustration, disengagement, or a diminished sense of control. These reactions are similar to the concept of LH, a psychosocial state characterized by passivity and diminished motivation following repeated experiences of uncontrollable difficulties [15,16]. LH arises when individuals attribute their failures to stable, global, and internal causes rather than to unstable, specific, and external ones, leading to the belief that they have no control over negative outcomes [15]. Consequently, individuals may avoid social activities that challenge this attributional style, resulting in behavioral disengagement and an increased risk of depression [17]. Psychosocial well-being refers to the presence of positive feelings or, in other words, the absence of anxiety and depression [18], and reflects an individual’s overall life satisfaction. In contrast, LH can be a debilitating condition that leads to depression, anxiety, hopelessness, and reduced social participation [17]. Although evidence regarding the effect of hearing loss on LH is limited, McCrone [19] reported elevated LH in deaf adolescents with academic difficulties, and Juneja et al. [20] showed that auditory rehabilitation reduced LH and improved quality of life in individuals with hearing aids.

Despite growing interest in psychosocial well-being, to our knowledge, no study to date has specifically investigated the LH phenomenon in CI users who are deaf or hard of hearing and depend entirely on their implants for auditory perception. In fact, CI users generally experience significant auditory benefit, which in turn supports their communication skills and engagement in everyday social activities [3,10]. However, considerable performance differences remain among CI users. Those implanted at an early age and provided with targeted rehabilitation in supportive environments tend to participate actively in social, academic, and professional contexts. However, others continue to face difficulties in complex everyday listening situations, with possible negative effects on their psychosocial well-being [21]. Understanding the prevalence and underlying auditory factors of LH in CI users may be beneficial for improving their psychosocial well-being and quality of life. Motivated by these considerations, the main goal of this study was to investigate the LH phenomenon among CI users and to compare their findings with those obtained from TH individuals. A further objective was to examine how LH relates to demographic, audiological, and psychosocial variables. Particular attention was given to everyday speech perception performance and to the presence and extent of LH effects in long-term CI users. The study also explored how auditory perception, particularly in noisy environments, and psychosocial well-being are linked to age at intervention within a subgroup of prelingually deaf young CI users implanted during childhood. It also sought to identify the speech-in-noise task that better reflects individual differences in real-world auditory perception and psychosocial adjustment. Insights into the relationship between everyday auditory performance and psychological state may help explain why some CI users achieve stronger emotional and social adjustment than others. Indeed, the psychosocial well-being of CI users should be regarded as a key indicator of long-term rehabilitation success.

## 2. Materials and Methods

### 2.1. Participants

Ethical approval was obtained from the local Ethics Committee (E-74555795-050.04-1257097). Written informed consent was obtained from all participants in accordance with the Declaration of Helsinki.

A power analysis performed using G*Power (version 3.1.9.2) determined that a minimum of 30 participants in each group would provide adequate statistical power (1 − β = 0.80) to detect a medium-to-large effect size (d = 0.75) at an alpha level of 0.05. The CI and TH groups comprised 60 adult native Turkish speakers in total (n = 30 per group), with mean ages of 22 ± 8 (range: 16–59) years and 22 ± 3 (range: 18–28) years, respectively. All but one of the CI users (who was 59 years old) were young adults. There was no significant difference in age between the two groups (*t*(58) = −0.285, *p* = 0.777). The mean age at implantation and duration of CI use in the CI group were 8 ± 10 years and 14 ± 6 years, respectively. In the present study, prelingual hearing loss was defined as hearing loss with an onset before 12 months of age, typically congenital or arising in infancy. In the prelingually deaf CI subgroup, the mean age at implantation and duration of CI use were 2 ± 1 years and 17 ± 2 years, respectively. The demographic details of the CI users are summarized in Table 1.

Exclusion criteria included neurological, psychiatric, or additional developmental disorders; auditory neuropathy spectrum disorder; cochlear nerve pathology; and recurrent middle ear disease. TH participants were required to have PTAs ≤ 20 dB HL between 0.25 and 8 kHz. The mean aided threshold was 26 ± 6 dB HL in the CI group, whereas the mean hearing threshold was 2 ± 4 dB HL in the TH group. Cognitive function was screened using the Montreal Cognitive Assessment (MoCA), which was developed by Nasreddine et al. [22] and adapted for the Turkish population by Selekler et al. [23].

### 2.2. Auditory Measures

Participants performed all laboratory tests in a double-walled soundproof booth. CI users were tested in their everyday listening mode, including unilateral, bilateral, or bimodal listening, and subsequently completed self-report measures of LH, depression, and anxiety.

#### 2.2.1. Hearing Thresholds

Pure-tone averages (PTAs) were calculated across 250, 500, 1000, 2000, 4000, 6000, and 8000 Hz. Measurements were obtained using an Otometrics Madsen^®^ Astera2 Clinical Audiometer (Ballerup, Denmark) with insert earphones. For CI users, aided sound-field thresholds were measured using a Logitech S120 loudspeaker (Lausanne, Switzerland) positioned at 0° azimuth and 1 m from the listener. Thresholds were determined using the modified Hughson–Westlake procedure in 5 dB steps.

#### 2.2.2. Speech Perception

Speech perception was assessed using the Hearing-in-Noise Test (HINT) [24,25] and the Turkish Matrix Sentence Test [26,27]. Both tests were developed to measure an individual’s ability to recognize sentences in both quiet and noisy listening conditions. Speech recognition in quiet conditions was calculated as the percentage of correctly repeated words, whereas speech-in-noise performance was quantified using the speech reception threshold (SRT), defined as the signal-to-noise ratio (SNR) corresponding to 50% recognition accuracy, with lower SRTs indicating better performance. Participants repeated each word they heard and received no feedback. Stimuli were presented in a sound-field through loudspeakers positioned at 0° azimuth and a distance of 1 m, with speech-shaped noise fixed at 65 dB SPL. A ceiling SNR of 20 dB was assigned to participants who showed no response, indicating a performance floor.

The Turkish version of the HINT comprises 12 lists of 20 sentences spoken by a male talker. The speech-shaped noise was spectrally matched to the long-term average spectrum of the speech material. Speech levels were adjusted adaptively, initially in 4 dB steps and subsequently in 2 dB steps, and the participant’s 50% SRT was calculated using the SNRs from sentences 5 through 20. Stimuli were delivered via a Logitech S120 loudspeaker (Lausanne, Switzerland). Calibration was performed using pink noise and a Larson Davis 824 sound-level meter (A-weighted, slow response) (Depew, NY, USA), adjusted to 90 dB SPL at ear level [25].

The Turkish Matrix Sentence Test consists of 50 commonly used Turkish words, including 10 names, 10 numerals, 10 adjectives, 10 objects, and 10 verbs, combined in a fixed syntactic structure (name + numeral + adjective + object + verb) to create semantically unpredictable yet grammatically correct sentences. Verbs were inflected with third-person singular and past-tense suffixes, whereas the remaining word categories were presented without inflection. Thirty lists of 20 sentences were available, and test lists were randomly selected without repetition for each participant. Stimuli were presented via a JBL Control One loudspeaker (JBL, Los Angeles, CA, USA). Target speech levels were adaptively varied in 1 dB increments, decreasing when word recognition exceeded 50% and increasing otherwise. The SRT was estimated using a maximum likelihood procedure, with speech levels dynamically adjusted according to performance on the preceding sentence [27].

#### 2.2.3. Hearing Implant Sound Quality Index

Sound quality perception was evaluated using the Hearing Implant Sound Quality Index (HISQUI). This tool was originally developed by Amann and Anderson [28]. The Turkish version demonstrated high internal consistency (α = 0.94) [29]. The HISQUI measures CI users’ auditory benefit across various everyday listening situations. It consists of 19 items, with ratings ranging from never (1) to always (7). The total score ranges from 19 to 133, and higher scores reflect greater auditory benefit: scores < 30 = very poor benefit, 30–59 = poor benefit, 60–89 = moderate benefit, 90–109 = good benefit, and 110–133 = very good benefit.

### 2.3. Psychosocial Measures

#### 2.3.1. Learned Helplessness Scale

LH was evaluated using the 20-item Likert-type Learned Helplessness Scale (LHS) developed by Quinless and Nelson [30]. The LHS was adapted into Turkish by Boysan [31], and the Turkish version demonstrated good internal consistency, with Cronbach’s α = 0.80. The scale uses a four-point rating system, ranging from “strongly agree” (1) to “strongly disagree” (4). Ten items are reverse scored for consistency; therefore, total scores range from 20 to 80. Higher total scores indicate greater levels of helplessness. The Turkish version of the scale comprises three validated factors: internality–externality, globality–spatiality, and stability–instability.

#### 2.3.2. Beck Depression Inventory

Depressive symptoms were measured using the Beck Depression Inventory (BDI), developed by Beck et al. [32]. The Turkish version was validated by Hisli [33], who confirmed its internal consistency, with Cronbach’s α = 0.80. The BDI comprises 21 items, each scored from 0 to 3. This produces a total score ranging from 0 to 63, with higher scores indicating greater symptom severity. Total scores of 0–9 indicate minimal depressive symptoms, 10–16 indicate mild symptoms, 17–29 indicate moderate symptoms, and 30–63 indicate severe symptoms.

#### 2.3.3. Beck Anxiety Inventory

Anxiety levels were assessed using the Beck Anxiety Inventory (BAI), developed by Beck et al. [34]. Ulusoy et al. [35] adapted the BAI into Turkish and confirmed its internal consistency, with Cronbach’s α = 0.93. The scale includes 21 items rated from 0 (not at all) to 3 (severely). Total scores range from 0 to 63. Scores of 0–7 represent minimal anxiety, 8–15 represent mild anxiety, 16–25 represent moderate anxiety, and 26–63 represent severe anxiety. A cut-off score of 16 suggests clinically significant anxiety.

### 2.4. Statistical Analysis

Data were analyzed using IBM SPSS Statistics v23. HINT and Matrix recognition scores in quiet conditions were converted to rationalized arcsine units (RAUs) to stabilize variance and minimize potential floor and ceiling effects [36,37]. The Shapiro–Wilk test indicated that age, PTAs, speech perception outcomes, psychosocial measures, and age at implantation were not normally distributed in at least one group. For this reason, non-parametric tests were applied. Between-group differences were examined using the Mann–Whitney U test, whereas within-subject comparisons were investigated using the Wilcoxon signed-rank test. Relationships among demographic, audiological, and psychosocial measures in the overall CI group (*n* = 30) were evaluated using Spearman’s rank-order correlation coefficient. The effects of age at implantation were further investigated in a subgroup of prelingually deaf long-term CI users implanted during childhood (*n* = 20). Only significant correlations were reported in the Results Section, and a cutoff value of *p* < 0.05 was used. Effect sizes were calculated using Rosenthal’s *r*, interpreted as small for values between 0.10 and 0.30, moderate for values between 0.30 and 0.50, and large for values of 0.50 or greater [38]. The strength of correlations was interpreted as very weak for values between 0.00 and 0.19, weak for values between 0.20 and 0.39, moderate for values between 0.40 and 0.59, strong for values between 0.60 and 0.79, and very strong for values between 0.80 and 1.00 [39].

## 3. Results

### 3.1. Speech Perception Findings

For clarity, speech recognition scores obtained under quiet listening conditions are presented as percentages in Table 2. RAU scores ranged from 12.78 to 112.78, corresponding to recognition accuracies between 0% and 100%. CI users performed significantly worse than the TH group on both the HINT (*U* = 18, *p* < 0.001, *d* = 0.98) and the Matrix Test (*U* = 35, *p* < 0.001, *d* = 0.95).

**Figure 1:**
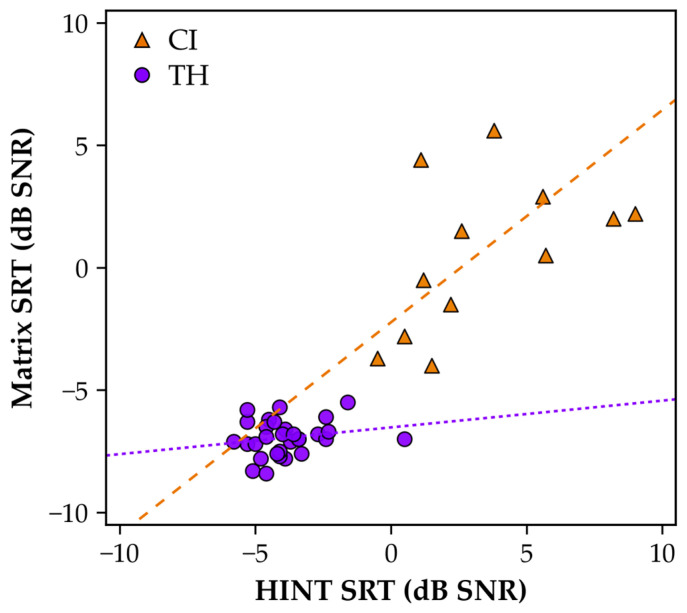
Individual speech-in-noise results on HINT and the Matrix tests. CI = Cochlear Implant, TH = Typical Hearing, HINT = Hearing-in-Noise Test, SRT = Speech Reception Threshold, dB SNR = Signal-to-Noise Ratio in decibels.

Speech-in-noise outcomes are illustrated in Figure 1 and Table 2, reflecting large intersubject variability among CI users. Significant between-group differences were observed for both HINT (*U* = 1.5, *p* < 0.001, *d* = 0.96) and Matrix (*U* = 0, *p* < 0.001, *d* = 0.98) SRTs, with CI users performing significantly worse than the TH group.

Within each group, HINT and Matrix outcomes were compared separately for quiet and noise conditions. No significant difference was observed between the two tests in the quiet condition (*p* > 0.05). In contrast, under speech-in-noise conditions, HINT SRTs were significantly higher than Matrix SRTs, indicating poorer performance on the HINT than on the Matrix test in both the CI group (*Z* = −3.3, *p* = 0.001, *d* = 0.70) and the TH group (*Z* = −4.8, *p* < 0.001, *d* = 0.88).

### 3.2. Psychosocial Findings

**Figure 2:**
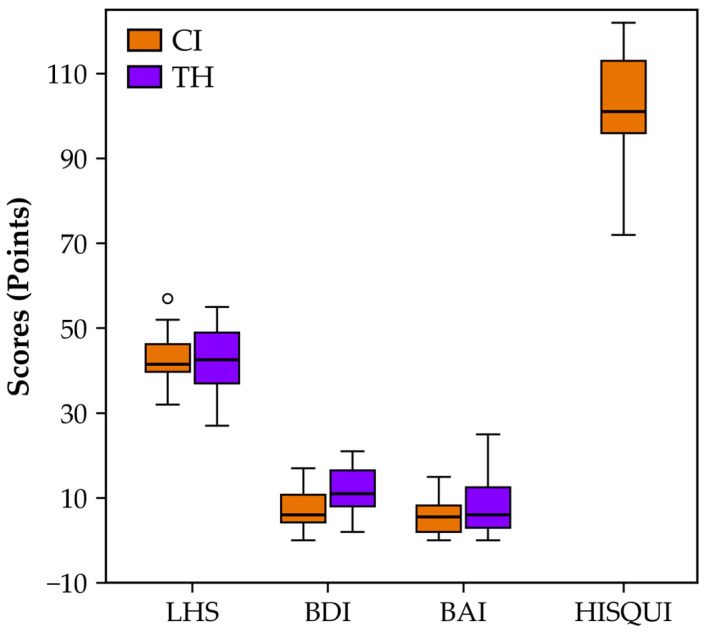
Psychosocial findings for the CI and TH groups. Boxplots represent scores on the LHS, BDI, BAI, and HISQUI. CI = Cochlear Implant, TH = Typical Hearing, LHS = Learned Helplessness Scale, BDI = Beck Depression Inventory, BAI = Beck Anxiety Inventory, HISQUI = Hearing Implant Sound Quality Index.

Psychosocial outcomes are presented in Figure 2 and Table 2. No significant between-group differences were found for LHS scores (*U* = 423.5, *p* = 0.695, *d* = 0.022), BDI scores (*U* = 395.5, *p* = 0.420, *d* = 0.051), or BAI scores (*U* = 443.5, *p* = 0.923, *d* = 0.014).

### 3.3. Correlational Findings in the Overall CI Group

HINT and Matrix scores showed strong positive correlations under both quiet (r_s_ = 0.65, *p* = 0.001) and noisy (r_s_ = 0.76, *p* < 0.001) listening conditions. Education level showed a moderate negative correlation with HISQUI scores (r_s_ = −0.42, *p* = 0.022).

LHS scores showed a moderate positive correlation with BDI scores (r_s_ = 0.45, *p* = 0.013). BDI and BAI scores were strongly positively correlated (r_s_ = 0.69, *p* < 0.001). BDI scores showed a moderate negative correlation with the duration of CI use (r_s_ = −0.53, *p* = 0.002).

### 3.4. The Effects of Age at Implantation

Within the prelingually deaf CI subgroup (*n* = 20), age at implantation was strongly positively correlated with aided PTAs (r_s_ = 0.66, *p* = 0.01). Earlier implantation was significantly associated with better speech recognition in quiet, as reflected by higher quiet-condition scores on both the HINT (r_s_ = −0.55, *p* = 0.04) and Matrix test (r_s_ = −0.69, *p* = 0.007). In addition, age at implantation was very strongly positively correlated with speech-in-noise SRTs on both the HINT (r_s_ = 0.85, *p* < 0.001) and Matrix test (r_s_ = 0.85, *p* < 0.001), indicating that later implantation was associated with poorer speech-in-noise performance. Overall, earlier implantation was significantly associated with better CI thresholds and superior speech perception performance.

## 4. Discussion

Cochlear implantation considerably improves communication skills and quality of life in people with severe-to-profound hearing loss [9]. Although speech perception in complex environments and subjective reports reflect persistent listening difficulties, CI users generally show significant improvement over time [14]. However, their well-known listening difficulties raise questions about whether these challenges might be linked to psychological patterns such as LH, as previously reported in deaf individuals, or whether the functional hearing (re)gained through a CI helps to minimize such psychosocial risks. The present study attempted to extend the limited body of research on hearing-impaired individuals by examining whether such patterns persist in CI users who, on the one hand, experience the restoration of functional hearing, yet on the other hand must still navigate complex auditory environments. In post-lingually deafened adults, hearing loss over the life course may precipitate LH; however, cochlear implantation offers the potential to counteract this effect by (re)establishing auditory perception and supporting active engagement with the environment. Indeed, the key question concerns which effect is more dominant: the benefits of (re)gained hearing or the challenges associated with complex listening demands. Overall, for the measures used in this study, the present sample of long-term CI users show psychosocial outcomes comparable to those of their TH peers. Although the cross-sectional design does not allow causal conclusions regarding the effects of cochlear implantation, these findings are consistent with previous studies suggesting that early intervention and sustained auditory access may support auditory, linguistic, cognitive, and emotional development [9]. Moreover, given that the majority of the present participants had prelingual deafness and long-term CI experience, it seems reasonable to suggest that early auditory access through cochlear implantation facilitates effective psychosocial integration into the auditory world, despite persistent difficulties in complex everyday listening situations.

The absence of significant group differences in LH, depression, or anxiety highlights the positive psychosocial outcomes that can be achieved through long-term auditory rehabilitation. Although previous studies have reported higher rates of depressive and anxiety symptoms in individuals with hearing impairment prior to implantation [10,17], improvements in self-esteem and emotional functioning after implantation are well documented [11]. The median LH scores for CI users (41.5) and TH peers (42.5) were within the previously reported normative range [17], indicating that CI users did not exhibit increased feelings of helplessness. Median BDI and BAI scores for CI users also fell within typical ranges, indicating that psychosocial well-being can be achieved with long-term CI use during late adolescence and adulthood.

The psychosocial findings in CI users indicate that established models linking hopelessness with emotional distress and decreased motivation are also valid for this patient group, as shown by positive correlations between LH and depressive symptoms and between depressive symptoms and anxiety [16,40]. In particular, despite poorer speech-in-noise performance reflecting everyday auditory perception compared with individuals with TH, normal psychosocial findings may be supported by adaptive communication strategies, such as effective coping mechanisms, the use of visual cues, contextual inference, and cognitive control of attention. These strategies have been shown to mitigate the emotional impact of listening difficulties. Thus, the present findings suggest that cochlear implantation can support social participation despite hearing impairment and may alleviate feelings of helplessness or frustration [15,16].

The HINT and Matrix tests are well-known speech perception tests that aim to reflect everyday auditory perception. Both tests are highly reliable and have been adapted for use in multiple languages, enabling cross-linguistic comparisons [25,26]. The Matrix Test is known to produce a learning effect, which is typically reduced after the second session. In contrast, the HINT consists of semantically meaningful everyday sentences [25]. To minimize contextual influence, the Matrix Test uses semantically unpredictable material [26,27]. As these tests capture different aspects of real-world listening, both were used to provide a more comprehensive view of auditory performance.

The present CI users showed significantly poorer speech-in-noise performance than individuals with TH, consistent with previous studies [14]. The HINT results were consistent with those of previous studies [25], with TH individuals achieving a median score of 100% for speech perception in quiet and −4.1 dB SNR for speech perception in noise. Similarly to the results reported by Cullen et al. [41], CI users achieved median scores of 78% and 5.3 dB for speech perception in quiet and noise, respectively. The Matrix Test outcomes were consistent with those reported by Dietz et al. [42], with TH individuals achieving a median score of 100% for speech perception in quiet and −7 dB SNR for speech perception in noise. Also, CI users achieved median scores of 80% and 2 dB in speech-in-quiet and speech-in-noise, respectively. These findings suggest that long-term CI users may achieve relatively good speech recognition in quiet conditions; however, the present results also revealed significant group differences in both quiet and noisy conditions, with CI users performing worse than TH individuals [43]. Nevertheless, the present results revealed significant group differences in both quiet and noisy conditions, with CI users performing worse than TH individuals. These results are largely consistent with the literature reporting that multiple speakers and/or competing noise are major problems for CI users [14]. These findings indicate that cochlear implantation successfully restores access to sound but also suggest that performance differences among individuals are determined by many factors, such as auditory experience, cognitive resources, and adaptation. The current findings emphasize the ability of the HINT and Matrix tests to reveal intersubject differences in everyday auditory performance, as well as their complementary roles.

As expected, the present HINT and Matrix outcomes showed strong correlations in both quiet and noisy listening conditions, despite substantial differences in the nature of the two tests. The HINT uses everyday sentences with semantic content, enabling listeners to use linguistic and contextual cues to facilitate comprehension. This characteristic is intended to closely resemble real-world communication environments [25]. Conversely, the Matrix Test involves word sequences with no semantic content, minimizing the effects of predictability. However, the closed-set nature of the test, involving a total of 50 words, generally allows CI users to perform better [12,27]. Indeed, the significant correlations between CI users’ performances on the HINT and Matrix tests may suggest that both measures capture related aspects of speech perception. However, speech perception performance differed between the two tests, with CI users performing significantly better on the Matrix test than on the HINT. This difference may stem from the closed-set nature of the Matrix test, whereas the HINT may place greater demands on sentence-level perception in a more real-life linguistic context. Therefore, these findings suggest that the two tests provide overlapping but complementary information about speech perception in CI users. Recent efforts to improve the ecological validity of speech perception assessments, including the simulation of realistic acoustic environments, competing background noises, and audiovisual cues, further support the need for complementary approaches when evaluating speech perception outcomes in CI users [13,14].

Despite poorer speech perception performance, CI users did not differ significantly from TH individuals in helplessness, depression, or anxiety scores. These findings indicate that, for the psychosocial measures used in the present study, long-term CI users showed outcomes comparable to those of TH individuals. However, pre-implantation psychosocial status was not assessed; therefore, the present data do not allow conclusions about whether these outcomes were caused by cochlear implantation or whether similar psychosocial profiles were already present before implantation. Rather, the findings should be interpreted as consistent with previous studies suggesting that early intervention and sustained auditory access may be associated with favorable psychosocial outcomes, while longitudinal studies are needed to determine causal effects [2,6].

The correlational findings observed within the CI group highlight the complex but close relationships among age at implantation, audiological outcomes, and psychosocial well-being. First, there was a moderate negative relationship between educational level and self-reported hearing quality within the overall CI group. These results may be related to personal and social expectations, as well as increasing demands and pressures from academic and/or professional life among individuals with higher education levels. Achieving higher education levels may reflect better auditory perception to some extent; however, sound quality expectations and related frustration may also be higher. Indeed, similar discrepancies have been shown between perceptual accuracy and music appraisal among CI users, suggesting that subjective reports of auditory experience may be affected by psychosocial factors, including personal and social needs, expectations, and related frustration [12]. As shown in Table 1, several CI users who were prelingually deaf but implanted at an early age successfully integrated into academic life and are pursuing careers in fields such as medicine, engineering, and media. These cases demonstrate the potential for academic success and social participation following effective long-term rehabilitation after early implantation.

Second, a moderate negative correlation was observed between BDI scores and the duration of CI use, indicating that longer CI experience was associated with fewer depressive symptoms. This finding suggests potential psychological benefits of prolonged CI use, possibly related to improved communication skills and social integration over time. Finally, a moderate negative correlation was found between speech-in-noise performance and aided sound-field PTAs obtained with the CI across octave frequencies from 250 Hz to 8 kHz. This finding indicates that better CI-aided auditory thresholds were associated with improved everyday auditory perception. This relationship supports earlier studies that limited PTA calculations to 0.25–4 kHz [5]. It also suggests that peripheral auditory sensitivity, as reflected by aided thresholds, may contribute to successful auditory perception in real-world listening conditions.

Furthermore, the present study performed correlational analyses in a subgroup of prelingually deaf CI users to investigate long-term outcomes, with a particular focus on the effects of age at implantation. In this subgroup, which represented the majority of the overall CI sample, significant effects of age at implantation were observed on CI thresholds and speech perception performance, highlighting the critical influence of early auditory access on long-term auditory outcomes [5,13]. Although no significant direct correlations were observed between auditory and psychosocial outcomes in the present study, this does not exclude the possibility of more complex relationships among these variables. Future studies with larger samples should examine whether auditory factors, such as age at implantation, aided thresholds, and speech perception performance, are indirectly associated with psychosocial outcomes through mediating variables such as language development, communication experience, educational background, or social participation.

The present insights into the effects of auditory (re)habilitation on psychosocial well-being are expected to encourage further research in CI users. Nevertheless, some limitations of this study should be acknowledged. Although no significant age differences were observed between CI and TH groups, and the single older CI participant (59 years old) showed a performance comparable to that of the younger CI users, the potential contribution of age-related auditory or cognitive factors should still be considered. Future studies with larger sample sizes and more age-balanced cohorts are needed to better characterize the effect of age on auditory and psychosocial outcomes in CI users. On the other hand, the cross-sectional design means that generalizations about the relationships between audiological characteristics and psychosocial measures should be interpreted with caution. Moreover, the sample size was calculated for group-level comparisons and was not adequate for statistical comparisons between prelingually deaf and post-lingually deafened CI users. In addition, self-report measures may underestimate subtle emotional difficulties or social adaptation challenges due to self-selection bias. Future research may adopt longitudinal designs to capture developmental trajectories of psychosocial states and integrate objective measures, such as electrophysiological analyses, to examine helplessness, depression, stress, and stress physiology under listening effort during speech-in-noise performance. Combining behavioral, electrophysiological, and self-report data would clarify the mechanisms through which auditory experience shapes social outcomes in CI populations. Moreover, individuals with superior cognitive abilities may be more adept at developing and using adaptive strategies to cope with communication difficulties and daily stressful situations. Such relationships should be investigated further in future studies using specific cognitive measures, such as verbal/nonverbal intelligence, memory, and attention [44]. This approach would improve our understanding of the mechanisms underlying psychological resilience and effective auditory rehabilitation.

## 5. Conclusions

Despite persistent difficulties with speech-in-noise perception, long-term CI users show levels of learned helplessness, depression, and anxiety comparable to those of TH individuals. Although pre-implantation psychosocial status was not assessed, these results suggest that psychosocial well-being can be maintained despite ongoing auditory challenges. A holistic approach considering longitudinal cognitive, social, psychosocial, and auditory outcomes may help to optimize overall quality of life in CI users.

## Figures and Tables

**Table 1:** Demographic data of the CI users.

			Number (n)	Percentage (%)
**Gender**		Woman	19	63.3
		Man	11	36.7
**Education Degree**		High School	23	76.7
		Bachelor’s Degree	6	20
		Master’s Degree	1	3.3
**Onset of Hearing Loss**		Prelingual	20	66.7
		Postlingual	10	33.3
**CI Brand**		Advanced Bionics	24	80
		MED-EL	6	20
**Processor**	Advanced Bionics	Naida CI Q90	17	56.7
		Naida CI Q70	3	10
		Naida CI Q30	1	3.3
		Neptune	3	10
	MED-EL	Rondo 2	5	16.7
		Sonnet 2	1	3.3
**Electrode Type**	Advanced Bionics	HiFocus 1J	20	66.7
		HiFocus Mid-scala	4	13.3
	MED-EL	Standard Compressed	5	16.7
			1	3.3
**Sound Coding Strategy**	Advanced Bionics	HiRes Optima-S	17	56.6
		HiRes Optima-P	2	6.7
		HiRes-S Fidelity 120	5	16.7
	MED-EL	FS4-P	5	16.7
		HDCIS	1	3.3

**Table 2:** Speech perception and psychosocial outcomes. Note. CI = Cochlear Implant, TH = Typical Hearing, HINT = Hearing-in-Noise Test, LHS = Learned Helplessness Scale, BDI = Beck Depression Inventory, BAI = Beck Anxiety Inventory, HISQUI = Hearing Implant Sound Quality Index.

		CI Group		TH Group				
		Median	Range	Median	Range	U	*p*	Effect Size
**Speech** **Perception**	HINT Quiet (%)	77.7	15–100	100	95–100	18	**<0.001**	**−0.98**
	HINT Noise (dB)	5.3	(−0.5)–20	−4.1	(−5.8)–0.5	1.5	**<0.001**	**−0.96**
	Matrix Quiet (%)	80	18–100	100	97–100	35	**<0.001**	**−0.95**
	Matrix Noise (dB)	2	(−4)–20	−7	(−8.4)–(−5.5)	0	**<0.001**	**−0.98**
**Psychosocial Measures**	LHS (score)	41.5	32–63	42.5	27–55	423.5	0.695	−0.02
	BDI (score)	9	0–32	11	2–21	395.5	0.420	0.05
	BAI (score)	7	0–33	6	0–25	443.5	0.923	−0.01
	HISQUI (score)	101	59–122	-	-	-	-	-

## Data Availability

The data presented in this study are available on request from the corresponding author due to ethical reasons.
